# Mental and Moral Might

**Published:** 1871-01

**Authors:** 


### HALL'S

### JOURNAL OF HEALTH.

Vol. XVIII. — JANUARY, 1871. — No. I.

### MENTAL AND MORAL MIGHT

Have their solid and deep foundations laid in a vigorous constitution and a healthy body. There are many men who are great, who have frail constitutions, or may become invalids; but in most cases, it will be found on examination that they had made their mark before they became so, or at least had founded their position while yet in good health. Among the men of renown of strong build may be named Cromwell, Webster, Washington, Franklin, Spurgeon, Beecher, John Hall, and others.

There is a proneness to select some quality in a great man's character upon which we can fix the reason of his greatness, and then to imagine that the practice of that quality will make the man who carries out that practice great. All imitators are weak-minded. Men of might feel their power, feel their sufficiency; their self-assertion is such that they would not stoop to be helped by practicing the peculiarity or the forte of another. The man apes his master, the sexton his clergyman, the maid her mistress, in proportion as the superior holds the inferior in awe; but most uniformly it is some peculiarity of a defective character which is imitated, such as a stutter, a lisp, a limp, or other fault or blemish. The only safe thing to imitate in the character of the great and good, and which copying is commendable, is the practice of their virtues. Not to go abroad, or to scatter the illustrations too widely, let the

CLERGY OF NEW YORK

be taken as examples of the principles in discussion. The metropolis of any country draws to it, with the certainty of the pole the magnet, the men of might, as well the evil as the good; and there such characters stay. A man may be called to a pulpit in the belief that he is a power, or will become so; but if the people have made a mistake, such a man will have to leave. It rarely happens that a great preacher leaves a great city; he dies at his post, unless in exceptional cases his health fails, or personal reasons induce a removal.

It may be well said that the pulpit of New York is a powerful pulpit; that it is filled by men of extraordinary abilities in every denomination, as McClintock, Duryea, Storrs, Cuyler, Beecher, Burchard, Hatfield, Paxton, Hall, Newell, "young Tyng," "Dr. Tyng," Montgomery, and others. All these are men who have might enough to collect and keep together large masses of men and women, old and young. There are other men of power in the pulpit here; but we speak only of those coming more particularly under our own personal notice and observation; saying nothing of the great and honored deeds of those who have been here, but have left, not for want of mental might or efficiency, or personal appreciation, but from motives of duty to the Church at large. No two of these men are "powerful" in the same thing; the secret of their might is not found in the combination of two or more qualities held alike by any two of them. Each one of them may be said to have power of his own, a power peculiar to himself, and which if a mere imitator were to attempt to exercise, he would most signally fail, because it is not natural to him; hence the effort to imitate is a loss of power of no mean amount; besides, the effort itself draws off the attention from the main point in hand, into which the entire personality should be thrown; for to succeed in anything, a man's whole soul should be thrown into it.

We "come at" a man most generally by the quality which strike us most forcibly; this quality is designated by the expressive word "peculiarity;" and in this sense it is intended to speak of several of the distinguished names here enumerated, in the order in which memory or association may bring them up.

"YOUNG TYNG,"

as he is familiarly called, is first noticed because his "church" is but a block or two away. He has a power surpassed by few living clergymen. Just seven years ago, one of the best of women as a wife, a mother, and friend, and of surpassing intelligence, sat down to a Sunday dinner, saying, "I have just heard a very remarkable sermon in a small room in the avenue." Her mind seemed so full of the sermon and the man, that the inquiry was made, —

"WHO IS HE?"

"He is a young man, and I think some one said he was a son of Dr. Tyng."

It was not long before that room could not hold half the people who wanted to come in. Later on, a "church" was built for him on some vacant outlots that seemed to stand a good chance of remaining vacant for a decade or two. That "church" is surrounded by the most elegant private mansions in the world. That "church" is one of the largest in membership in its denomination in America. In that "church" there cannot be found a single vacant sitting, and each pew rents for more a year than would pay the salary of many a worthy minister twenty years ago. That "church" gives more money, does more personal Christian and charitable labor, than any one of its age or material in all this wide country. Wherein lies the success of "young Tyng," a man not much over five and a half feet high, slim, weighing scarce a hundred and thirty pounds, frail in appearance, sandy hair, long side-whiskers, and in no way of imposing appearance? He has nothing of what is usually meant by eloquence of diction or oratorical power; he makes no flights of fancy, but he has a greater "make" than this by far. He has the power to

"MAKE HIS PEOPLE WORK."

His is emphatically a working church. He speaks with authority; and, like his old Roman dad, he fears no man. The Bible is the power behind the throne, and he never propounds a duty that he cannot follow up with a "Thus saith the Lord." He magnifies his office. He seems to consider himself an ambassador from the court of Heaven; that he has a right to be heard, and thereupon demands a hearing. He says to the banker, "If you do not give your money to help men to get to heaven, you are not a follower of Christ;" and when the plates go round, they return filled.

If he wants teachers for his constantly increasing Sunday-schools, he has only to announce it from the pulpit, and on next Sunday there are more applicants than are needed. If visitors are required to find out the sick and carry necessities to the poor and wretched, he says that man or woman who has no time to help the needy and the suffering can have no claim on His salvation who, when He was a sojourner on earth, went about doing good, saying to every human helper, "Inasmuch as ye did it to one of the least of these, ye did it unto me; enter ye into the joys of your Lord."

It was said that Mr. Tyng "spoke as with authority." This is an important and much needed element of ministerial success; the absolutely essential material requisite for which is an abundant salary. No minister can be morally brave who cannot practically observe the mandate —

"OWE NO MAN ANYTHING."

A man who cannot feel and assert his manhood in all places, and at all times, and before all persons, is not in a position to magnify his office. The clergyman who is so poorly paid that he must be in arrears to his baker, his butcher, his milkman, his tailor, has half the tools of his efficiency taken from him, and is compelled to make brick without straw.

When "young Tyng" leaves the church after the services are over, you can see by the manner in which the merchant prince, the banker, and the capitalist speak to him that they consider this slim, little, sandy-haired, frail, human specimen their superior. Mr. Tyng has a courtly address, which combines dignity, refinement, and respectful cordiality. In his whole manner he respects every man, and every man respects him, — respects him for his moral courage, for his talents, for his zeal, for his energy, and for his unblemished personal character. In short, —

He works himself for the Master, and makes others work by saying, " Follow me."

He gives himself and makes others give, because he makes them see that it is but "giving to the Lord," — to that Lord who "gave himself for us."

He has the gift of self-assertion, in that he feels himself a man, a peer to any of woman born, aside from the fact that in his official capacity he is second to none on mortal thrones.

Other names mentioned, and the secret of their power, will be handled hereafter.

